# The genome assembly of asparagus bean, *Vigna unguiculata* ssp. *sesquipedialis*

**DOI:** 10.1038/s41597-019-0130-6

**Published:** 2019-07-17

**Authors:** Qiuju Xia, Lei Pan, Ru Zhang, Xuemei Ni, Yangzi Wang, Xiao Dong, Yun Gao, Zhe Zhang, Ling Kui, Yong Li, Wen Wang, Huanming Yang, Chanyou Chen, Jianhua Miao, Wei Chen, Yang Dong

**Affiliations:** 1 BGI Education Center, University of Chinese Academy of Sciences, Shenzhen, 518083 China; 2 BGI Institute of Applied Agriculture, BGI-Shenzhen, Shenzhen, 518120 China; 3 Hubei Province Engineering Research Center of Legume Plants, College of Life Sciences, Jianghan University, Wuhan, 430056 China; 4 Center for Ecological and Environmental Sciences, Key Laboratory for Space Bioscience & Biotechnology, Northwestern Polytechnical University, Xi’an, 710072 China; 5 College of Biological Big Data, Yunnan Agricultural University, Kunming, 650201 China; 6 State Key Laboratory of Genetic Resources and Evolution, Kunming Institute of Zoology, Chinese Academy of Sciences, Kunming, 650223 China; 7 BGI-Shenzhen, Shenzhen, 518083 China; 8 Guangxi Key Laboratory of Medicinal Resources Production and Genetic Improvement, Guangxi Botanical Garden of Medicinal Plants, Nanning, 530023 Guangxi China; 9 National & Local Joint Engineering Research Center on Germplasm Utilization & Innovation of Chinese Medicinal Materials in Southwestern China, Kunming, 650201 China; 10 Key Laboratory of Genomics,Ministry of Agriculture, BGI-Shenzhen, Shenzhen, 518083 China

**Keywords:** DNA sequencing, Plant genetics

## Abstract

Asparagus bean (*Vigna*. *unguiculata* ssp. *sesquipedialis*), known for its very long and tender green pods, is an important vegetable crop broadly grown in the developing Asian countries. In this study, we reported a 632.8 Mb assembly (549.81 Mb non-N size) of asparagus bean based on the whole genome shotgun sequencing strategy. We also generated a linkage map for asparagus bean, which helped anchor 94.42% of the scaffolds into 11 pseudo-chromosomes. A total of 42,609 protein-coding genes and 3,579 non-protein-coding genes were predicted from the assembly. Taken together, these genomic resources of asparagus bean will help develop a pan-genome of *V*. *unguiculata* and facilitate the investigation of economically valuable traits in this species, so that the cultivation of this plant would help combat the protein and energy malnutrition in the developing world.

## Abstract (summary)

**Table:** 

Design Type(s)	sequence assembly objective • sequence annotation objective
Measurement Type(s)	genome assembly
Technology Type(s)	DNA sequencing
Factor Type(s)	
Sample Characteristic(s)	Vigna unguiculata subsp. sesquipedalis • leaf

Machine-accessible metadata file describing the reported data (ISA-Tab format)

## Background & Summary

Asparagus bean (*Vigna unguiculata* ssp. *sesquipedialis*, 2n = 2× = 22) is a warm-season and drought-tolerant subspecies of cowpea (*Vigna unguiculata*) with a wide cultivation area in East and Southeast Asia^1^. This plant is also known as yardlong bean because of its characteristic pod that grows up to 50–100 cm in length^2^. The long pod trait is believed to be the result of intensive local domestication after it was brought to Asia from sub-Saharan Africa^3^. Unlike the grain-type subspecies common cowpea (*Vigna*. *unguiculata* ssp. *unguiculata*, or black-eyed pea), asparagus bean is harvested while its pod is still tender, thereby providing a very good source of protein, minerals, vitamins, and dietary fiber^4^. Due to the low requirement for cultivation management and its high nutritional value, asparagus bean is one of the top crops that help combat malnutrition and food insecurity in most developing countries^5^.

As the DNA sequencing technologies became more advanced and affordable for the past decade, previous research had mainly focused on delineating the genome of common cowpea (estimated genome size of 620 Mb^6^). The first study of cowpea genomics was reported in 2008, in which the gene-rich space of cowpea was sequenced and assembled into 52,149 assemblies (41,260 assemblies were annotated) and 70,679 singletons^7^. Then the common cowpea (variety IT97K-499-35) genomic resources including a partial 323 Mb whole-genome shotgun assembly^8^, a 497 Mb bacterial artificial chromosome physical map^8^, and consensus genetic maps based on either 10 K^9^ or 50 K single nucleotide polymorphisms (SNPs) were available^8^. A more recent research reported two survey genomes of common cowpea (varieties IT97K-499-35 and IT86D-1010) with substantially improved assembly sizes (568 Mb and 609 Mb, respectively)^10^. In addition, a draft IT97K-499-35 variety reference genome was assembled by incorporating the single molecule real-time technology, yielding an assembly size of 519.4 Mb into 722 scaffolds and 11 pseudo-chromosomes^11^. Three genetic maps were derived from either simple sequence repeat markers^12,13^ or restriction-site associated DNA sequencing for asparagus bean^14^. Most of these genetic resources are focus on the grain-type cowpea, but there are many differences between the two types of cowpea, such as morphology, growing environments and parts for use^12^.

**Fig. 1:**
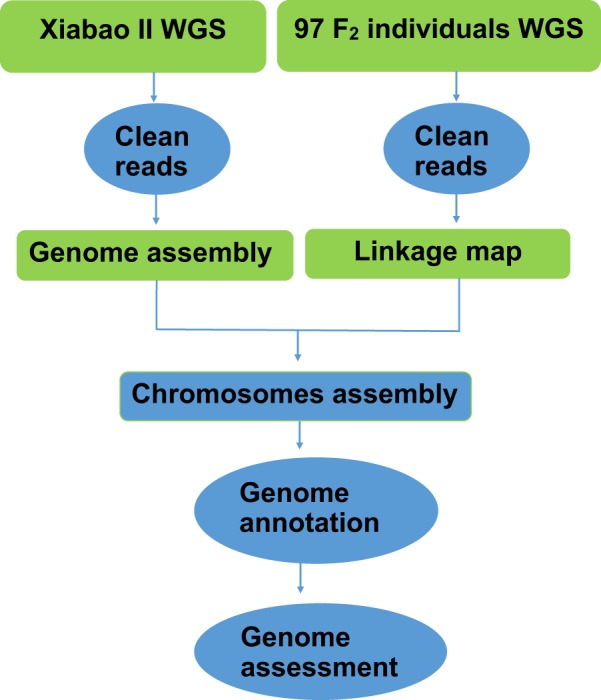
General description of the assembly workflow. The pipeline included removal of low quality and adapter-contaminated reads, *de novo* assembly, construction of linkage map, chromosome-scale assembly, and genome annotation.

**Fig. 2:**
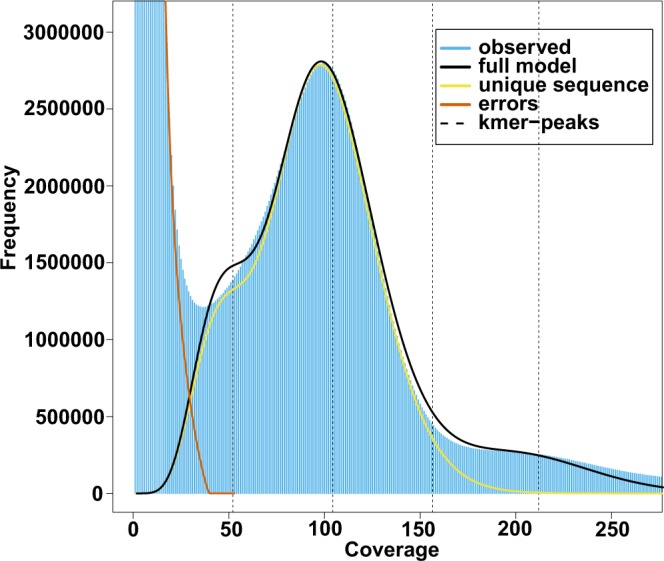
17-mer frequency distribution of sequencing reads.

In this study, we aimed to fill the knowledge gap with regard to the asparagus bean genome and provide new genetic resources for breeding cowpea and related legume species. A schematic workflow of the research is shown in Fig. 1. In brief, a series of short-insert and large-insert Illumina libraries were sequenced on an Illumina HiSeq 4000 platform, yielding a total of 222.9 Gb clean data (Table 1). Since the genome size of asparagus bean was estimated to be about 590 Mb using the *K*-mer distribution analysis (Table 2) (Fig. 2), the clean data used for genome assembly represented about 340× coverage. The software SOAPdenovo^15^ was used to generate a draft contig assembly of 549.8 Mb with a contig N50 size of 15.2 kb (Table 3). After scaffolding and gap closing, the final asparagus genome was 632.8 Mb (549.81 Mb non-N size) in size with scaffold N50 size of 2.7 Mb (Table 3). We also obtained 536,824 high-confident SNPs from the whole-genome sequencing data of 97 asparagus bean F2 individuals and two parents from a well-controlled selfing population. These SNPs were used to construct a high-density genetic map for asparagus bean, in which 1,556 scaffolds were successfully anchored onto 11 pseudo-chromosomes (Table 4). Furthermore, the asparagus bean genome contained 294.95 Mb of transposable elements, accounting for 46.47% of the assembly (Tables 5 and 6). The gene prediction was performed on a combination of *de novo*, homologous, and RNA-Seq-based approaches. It resulted in 42,609 protein-coding genes and 3,579 non-protein-coding genes, respectively (Table 7).

**Table 1:** Statistics of Raw Data after Filtering.

Insert Size	Clean Length (bp)	Number of Clean Reads	Clean Bases (Gb)	Sequence Coverae (X)
350	2 × 125 #Hiseq 4000	143,324,095	35.831	54.92
445	2 × 125 #Hiseq 4000	200,584,850	50.146	76.86
758	2 × 125 #Hiseq 4000	60,211,855	15.053	23.07
912	2 × 125 #Hiseq 4000	113,659,706	28.415	43.55
2000	2 × 125 #Hiseq 4000	79,141,602	19.785	30.32
3000	2 × 125 #Hiseq 4000	82,610,562	20.653	31.65
5000	2 × 125 #Hiseq 4000	80,415,362	20.104	30.81
9000	2 × 125 #Hiseq 4000	72,037,228	18.009	27.6
15000	2 × 125 #Hiseq 4000	59,701,495	14.925	22.87
Total	—	891,686,755	222.921	341.66

**Table 2:** Estimation of genome size and heterozygosity of asparagus bean by k-mer analysis.

k	Total number of k-mers	Minimum coverage (X)	Number of erroneous k-mers	Homozygous peak	Estimated genome size (Mb)	Estimated heterozygosity (%)
17	61,995,624,762	36	2,614,930,973	100	593.81	0.81758
19	61,069,619,958	30	5,112,996,411	93	601.68	0.90512
21	60,143,656,148	28	6,464,570,689	90	596.43	0.89166
23	59,217,725,396	27	7,226,388,959	89	584.17	0.83246
25	58,291,828,145	26	7,766,008,145	86	587.51	0.76826
27	57,365,972,074	26	8,206,249,053	84	585.23	0.71033
29	56,440,164,790	24	8,580,620,676	82	583.65	0.66007
31	55,514,382,010	22	8,908,657,517	79	589.95	0.61617

**Table 3:** Results of the asparagus bean genome assembly.

	Contigs		Scaffolds	
	Size (bp)	Number	Size (bp)	Number
N90	4,293	36,621	221,483	308
N80	7,053	26,804	918,008	183
N70	9,566	20,138	1,507,419	130
N60	12,222	15,059	2,195,354	96
N50	15,154	11,022	2,730,264	70
Longest	119,701	—	14,145,393	—
Total Number (> = 500b)	—	61,962	—	9,083
Total Number (> = 1 kb)	—	54,864	—	5,621
Total	549,819,688	80,696	632,812,756	21,836

**Table 4:** Statistics of pseudo-chromosomes and genetic map in asparagus bean.

Chromosomes	Anchored Scaffolds Number	Total length (Mb)	SNP Number	bin marker Number	Genetic distance (cM)	Gene Bank accession
Vu01	162	52.07	54,989	159	113.72	CP039350
Vu02	81	41.88	41,888	170	125.31	CP039348
Vu03	161	82.25	58,426	306	398.24	CP039346
Vu04	233	55.8	40,719	175	185.51	CP039349
Vu05	87	60.58	31,849	171	83.74	CP039354
Vu06	97	45.38	36,916	154	94.72	CP039345
Vu07	81	51.81	23,748	189	207.05	CP039353
Vu08	148	49.22	44,186	193	333.04	CP039351
Vu09	79	53.94	27,657	179	260.14	CP039355
Vu10	203	49.61	95,735	155	164.48	CP039352
Vu11	224	54.99	80,711	162	214.19	CP039347
Total	1556	597.53	536,824	2013	2180.14

**Table 5:** Statistics of Repeats in the asparagus bean genome.

Type	Repeat Size (bp)	% of genome
Trf	67,718,076	10.67
Repeatmasker	41,222,404	6.49
Proteinmask	64,741,265	10.2
*De novo*	264,487,557	41.67
Total	294,953,638	46.47

**Table 6:** TEs Content in the assembled asparagus bean genome.

Type	Repbase TEs		TE proteins		De novo		Combined TEs	
	Length (bp)	% in genome	Length (bp)	% in genome	Length (bp)	% in genome	Length (bp)	% in genome
DNA	6,870,914	1.0825	9,850,112	1.5518	41,887,195	6.5992	46,098,143	7.2626
LINE	698,393	0.11	1,195,466	0.1883	1,651,447	0.2601	2,666,968	0.4201
SINE	30,804	0.0048	—	—	62,452	0.0098	74,704	0.0117
LTR	33,989,514	5.3549	53,894,224	8.4908	112,113,184	17.6631	122,145,625	19.2437
Other	13,118	0.002	—	—	—	—	13,118	0.002
Unknown	—	—	—	—	119,021,287	18.7515	119,021,287	18.7515
Total	41,222,404	6.494	64,741,265	10.1998	261,567,318	41.2092	272,160,906	42.8782

**Table 7:** Prediction of protein-coding genes in asparagus bean genome.

Gene set		Gene number	Ave. gene length	Ave. CDS length	Total Exon number	Ave. exon number	Ave. exon length	Total intron number
Homology	Augustus	45,883	2,243.10	1,005.11	207,693	4.53	222.05	56,802,940
	Arabidopsis	26,867	3,133.37	1,080.92	124,326	4.63	233.59	55,143,207
	Pigeonpea	44,018	3,055.98	996.71	169,707	3.86	258.52	90,644,666
	Chickpea	29,722	3,267.60	1,101.41	135,727	4.57	241.19	64,383,299
	Soybean	35,380	2,919.91	1,032.92	152,214	4.3	240.09	66,761,546
	Lotus	37,713	2,436.51	912.21	142,619	3.78	241.22	57,486,204
	Medicago	37,164	2,785.79	951.18	148,495	4	238.05	68,181,528
	Rice	25,956	2,971.76	1,010.14	112,815	4.35	232.41	50,915,754
	Common bean	32,860	3,059.37	1,099.25	149,363	4.55	241.84	64,409,431
	Mungbean	29,468	3,695.35	1,123.44	143,184	4.86	231.21	75,789,153
	Grape	27,358	3,732.39	1,059.30	134,163	4.9	216.01	73,130,296
	Adzuki bean	37,596	3,191.78	991.8	160,449	4.27	232.4	82,710,459
Denovo	Genscan	40,736	8,880.46	1,153.45	230,011	5.65	204.28	314,767,263
	GlimmerHMM	46,755	1,867.51	847.52	164,690	3.52	240.61	47,689,651
Transcriptome		114,947	8,244.23	752.27	243,192	2.12	355.57	861,179,063
EVidenceModeler		42,609	3,156.05	1,043.18	190,304	4.47	233.57	90,027,213

## Methods

### Materials

All plant accessions were provided by Hubei Natural Science Resource Center for Edible Legumes in Wuhan of China. A single plant of the widely cultivated asparagus bean variety ‘Xiabao II’ (*Vigna unguiculata* ssp. *sesquipedialis* var. ‘Xiabao II’) was used for *de novo* sequencing and genome assembly. A F2 sequencing population was obtained for making the genetic map according to the following procedure. First, the F1 population were obtained by crossing ‘Xiabao II’ (male, same plant used for *de novo sequencing*) with a cultivar from the other subspecies, ‘Duanjiangdou’ (*Vigna unguiculata* ssp. *unguiculata* var. ‘Duanjiangdou’; female). This step yielded 17 seeds, from which only 12 seeds survived till flowering. These F1 individuals were bagged to promote selfing, which produced 561 seeds in total (the F2 generation). Only 367 of the F2 individuals were able to germinate and mature into full plants. We selected 97 of the 367 F2 individuals for genome sequencing and genetic map construction.

### Whole-genome shotgun sequencing

Young leaves were collected from a single ‘Xiabao II’ plant and used for genomic DNA extraction by the CTAB method^16^. About 10 µg of genomic DNA were used for library construction. Four short-insert libraries (350 bp, 445 bp, 758 bp, and 912 bp) and five large-insert libraries (2 kb, 3 kb, 5 kb, 9 kb, and 15 kb) were constructed with NEBNext Ultra II DNA Kit (NEB, America) and Nextera Mate Pair Sample Preparation Kit (Illumina, America), respectively. These libraries were sequenced on an Illumina HiSeq 4000 platform. To ensure high-quality reads for the subsequent *de novo* assembly step, we filtered out the low-quality data by the following criteria: (a) reads with >2% unidentified nucleotides (N) or with poly-A structure; (b) reads with ≥40% bases having low quality for short insert-size libraries and ≥60% for large insert-size libraries; (c) reads with adapters or PCR duplication; (d) reads with 20 bp in 5′ terminal and 5 bp in 3′ terminal. Subsequently, about 222.9 Gb clean data were retrieved^17,18^, covering 341.66-fold of the estimated genome (Table 1).

The genomic DNA was extracted with the same procedure for the parents and all 97 F2 individuals in the resequencing population. Each DNA was used to construct 500 bp insert size libraries, which were then sequenced on an Illumina HiSeq 4000 platform. Each individual was sequenced to at least 4× coverage. NGSQCToolkit_v2.3.3^19^ was used to filter low-quality reads (parameters: −l 70 −s 25) and trim the poor-quality terminal bases (parameters: −l 5 −r 5). A total of 882.67 Gb clean bases were kept, which represented 99% of the raw sequencing data^17,18^.

### Estimation of the genome size

The genome size of asparagus bean was estimated by the *k*-mer analysis approach using 69.42 Gb filtered short-insert sequencing data. The number of effective *k*-mers and the peak depth of a series of *k* values (17, 19, 21, 23, 25, 27 29 and 31) were generated by Jeffyfish (v2.2.6)^20^ with the C-setting and the genome size was estimated to be about 590 Mb, according to the formula Genome_Size = (Total *k*-mers - Erroneous *k*-mers)/Peak_depth (Table 2). It is worth noting that this number could be an underestimate, in that the GC rich regions and repetitive sequences could not be properly resolved by *k-*mer analysis. Nonetheless, our estimated genome size was within the range of previously reported sizes (560.3 Mb^11^~620 Mb^6^). The rate of genome heterozygosity was calculated with the *k*-mer frequency distribution by the GenomeScope (v1.0.0)^21^ and the result was around 0.77% (Fig. 2).

### *De novo* genome assembly

Clean data from short insert-size libraries were corrected with the Error Correction program in SOAPdenovo package^15^. Genome assembly was performed based on the *de Bruijn* graph algorithm using SOAPdenovo package^22^ by the following steps: (1) the paired-end reads of all libraries were used to construct the contig sequences while the *K*-mer values were set as 95 and 85 at the pregraph step and map step, respectively; (2) mapped paired reads were used to construct scaffolds; (3) The GapCloser package was used to map reads to the flanking sequences of gaps and to close gaps between the scaffolds; (4) genome sequence was randomly broken to re-scaffold with SSPACE package. Gaps were then filled again by GapCloser to obtain the final assembly. In the end, there were 54,864 out of 80,696 contigs with sizes longer than 1 kb. The total length of the contig assembly was 549.81 Mb (Table 3). The longest scaffold was 14,145,393 bp, and a total of 5,621 scaffolds were longer than 1,000 bp^17,23–25^. The total length of the scaffold assembly was 632.8 Mb (Table 3).

### High-density genetic map construction and genome assembly anchoring

All clean data obtained from the two parents and the 97 F2 individuals were mapped to the asparagus bean scaffold assembly using the Burrows-Wheeler-Alignment tool (BWA)^26^ mem algorithm. The SAM files were converted to BAM files using SAMtools^27^. Then the bam files were used to call SNP by the GATK software package^19^ with parameters “-T HaplotypeCaller -stand_call_conf 30.0 -stand_emit_conf 10.0” and “-T SelectVariants -selectType SNP”. The SNPs were filtered using GATK with parameters as the following:–filterExpression “QD <2.0 || ReadPosRankSum <−8.0 || FS >60.0 || MQ <40.0 || SOR >3.0 || MQRankSum <−10.0 || QUAL <30” –logging_level ERROR–missingValuesInExpressionsShouldEvaluateAsFailing. After genotyping, the raw SNPs were filtered with the following criteria: missing rate <0.3 and heterozygous genotypes <0.5, resulting in a total of 836,933 high-confidence SNPs^23^.

**Fig. 3:**
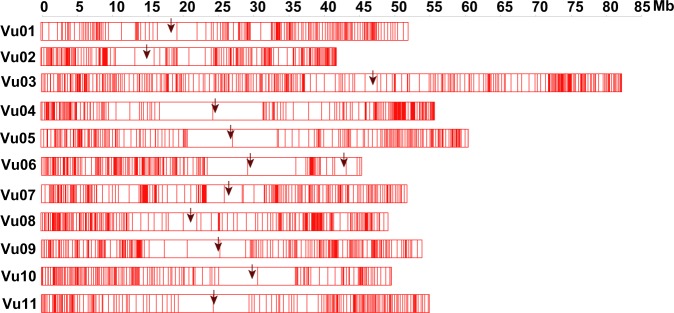
The distribution of bin markers. Black arrows indicated the low-recombination regions.

For the genetic map construction, 50 SNPs were selected to generate bin markers from the two termini and middle part of each scaffold. These bin markers were grouped into 11 linkage groups by JoinMap v4.1^28^ with the regression mapping algorithm. The grouped bins were then sorted and genetic distance was calculated by MSTmap with the Kosambi model^29^. According to this linkage map, scaffolds were anchored onto 11 pseudo-chromosomes. The SNPs were then assigned chromosome positions and a sliding window method (window size of 50 SNPs; step size of one SNP) was adopted to identify recombination events for each individual. All the recombination sites were merged and sorted with 20 kb intervals^30^. In the end, the filtered 536,824 SNPs^23^ were combined into 2,013 bins^23^. According to the distribution of bins, low-recombination regions were indicated (Fig. 3). These were used to construct 11 linkage maps, resulting in 2180.14 cM spanning the whole genome^23^, which was within the reported genetic map size ranged from 643 cM^31^ to 2,670 cM^32^. It is worth noting that the map lengths could be inflated by genotyping errors, as well as some biological phenomena causing double recombinations^33^. The sliding window method and bin markers were used to reduce genotype errors. Since the parents were not 100% homozygous, the F1 plants were not identical, which might result inflated genetic size. In addition, 1,556 scaffolds with 597.52 Mb were anchored^23^, accounting for 94.42% of the assembled genome (Table 4).

### Transposable elements annotation

Transposable elements (TEs) annotation were performed by a combination of homology-based and *de novo* prediction approaches. Homology-based approach involved searching commonly used databases for known TEs at both DNA and protein level. With default parameters, RepeatMasker 3.3.0^34^ was used to identify TEs against the Repbase TE library 18.07^35^ and RepeatProteinMask^34^ was used to identify TEs at the protein level in the genome assembly. For *de novo* prediction, RepeatModeler software (http://www.repeatmasker.org/) was used in constructing the *de novo* repeat library. Tandem repeats were then predicted by TRF^36^ with parameters set to “Match = 2, Mismatch = 7, Delta = 7, PM = 80, PI = 10, Minscore = 50 and MaxPeriod = 2000”. In total, we identified 294.95 Mb of the transposable elements, accounting for 46.47% of the asparagus bean genome (Tables 5 and 6). Among all TEs, long terminal repeat (LTR), which are important determinants of angiosperm genome size variation, constituted 19.24% of the assembled genome. DNA TEs accounted for 7.2% of the total sequence.

### Gene annotation

We used *de novo*, homology and RNA-Seq-based prediction methods to annotate protein-coding genes in the asparagus bean genome. Three *de novo* prediction programs, Augustus^37^, Genscan^38^ and GlimmerHMM^38^ were used to annotate protein-coding genes while gene model parameters were trained from *Arabidopsis thaliana*. For homology-based prediction, protein sequences of all the protein-coding genes of eleven species including common bean (*Phaseolus vulgaris*), soybean (*Glycine max*), pigeonpea (*Cajanus cajan*), chickpea (*Cicer arietinum*), mungbean (*Vigna radiata*), adzuki bean (*Vigna angularis*), lotus (*Lotus japonicus*), medick (*Medicago truncatula*), Arabidopsis (*Arabidopsis thaliana*), grape (*Vitis vinifera*), and rice (*Orzya sativa*), were first mapped to the asparagus bean genome using TblastN with the parameter E-value = 10^−5^. GeneWise^39^ was then used to predict gene structure within each protein-coding region. RNA-Seq data of root and stem tissues^40^ were aligned to the asparagus bean genome using TopHat on default settings. Finally, the predicted genes were merged by EvidenceModeler (EVM)^41^ to generate a consensus and non-redundant gene set. This process produced 42,609 protein-coding genes with an average length of 3,156 bp (Table 7).

With BLASTP (E-value ≤ 10^−5^), gene functions were assigned according to the best hit of alignment to SwissProt^42^, TrEMBL^43^, and KEGG^44^ database. Functional domains and motifs of asparagus bean genes were determined by InterProScan^45^, which analyzed peptide sequences against protein databases including SMART, ProDom, Pfam, PRINTS, PROSITE and PANTHER. Gene Ontology (GO) terms for each gene were extracted from the corresponding InterPro entries. The result showed that 75.40% (32,126) of the total genes were supported by TrEMBL, 56.22% (23,953) by Swiss-Prot, and 59.27% (25,254) by InterPro. In addition, 10,096 (23.69%) genes could not be functionally annotated with current databases (Table 8).

**Table 8:** Functional annotation of predicted genes in asparagus bean genome.

	Number	Percent (%)
Total	42,609	—–
InterPro	25,254	59.27
GO	19,254	45.19
KEGG	18,372	43.12
Swiss-Prot	23,953	56.22
TrEMBL	32,126	75.4
NR	32,356	75.94
Annotated	32,513	76.31

The tRNA genes were identified by tRNAscan-SE software^46^ with default parameters. The rRNA genes were identified based on homology search to previously published plant rRNA sequences using BLASTN with parameters of “E-value = 10^−5^”. The snRNA and miRNA genes were identified by INFERNAL v1.0^47^ software against the Rfam database with default parameters. In all, 3,579 non-protein-coding genes were identified in the asparagus bean genome, including 1593 tRNAs, 1,076 rRNAs, 350 snRNAs, and 210 microRNAs (Table 9).

**Table 9:** Annotation of non-coding RNA in asparagus bean genome.

Type		Copy(w)	Average Length(bp)	Total Length(bp)	% of genome
miRNA		210	118.0571	24792	0.003906
tRNA		1593	75.10295	119639	0.018849
	rRNA	538	155.6636	83747	0.013194
	18S	114	346.0877	39454	0.006216
rRNA	28S	77	116.2338	8950	0.00141
	5.8S	22	146.8636	3231	0.000509
	5S	325	98.80615	32112	0.005059
	snRNA	350	120.44	42154	0.006641
snRNA	CD-box	179	102.1285	18281	0.00288
	HACA-box	24	123.7083	2969	0.000468
	splicing	147	142.2041	20904	0.003293

## Data Records

The authors declare that all data reported here are fully and freely available from the date of publication. The data resulting from each experimental and analytic step are indicated in a table (Table 10). The assembly genome and annotation are available at CNSA^17^, figshare^23^, GenBank and have accessions CP039345^24^ to CP039355^25^. Raw read files of genome sequencing are available at NCBI Sequence Read Archive^18^ and CNSA^17^. The SNP sets of each pseudo chromosome, the anchored scaffolds information, the filtered SNPs set identified by GATK, the information of bin markers and the linkage map constructed by bin markers are deposited in figshare^23^. The RNA-seq data was deposited in figshare^40^.

**Table 10:** Experimental study and data records.

Subjects	Protocol 1	Protocol 2	Protocol 3	Data 1		Protocol 4	Data 2	
Xiabao II	Young leaves dissection	DNA extraction	Whole-genome shotgun sequencing	https://identifiers.org/ncbi/insdc.sra:SRP144706	Accession range: SRR7135464-SRR7135488	*De novo* genome assembly and annotation	https://www.ncbi.nlm.nih.gov/nuccore	Accession range: CP039345-CP039355
							https://db.cngb.org/search/?q=CNP0000264&from=CNSA	
							10.6084/m9.figshare.8131823	
97 F2 individuals	Young leaves dissection	DNA extraction	Whole-genome resequencing	https://identifiers.org/ncbi/insdc.sra:SRP144706	Accession range: SRR7125688-SRR7125784	Genetic map construction and chromosome assembly	10.6084/m9.figshare.8131823	
Xiabao II	Root and stem tissues	RNA extraction	RNA-seq	10.6084/m9.figshare.8131535		Annotation based on RNA-seq	10.6084/m9.figshare.8131823	

## Technical Validation

### DNA sample quality

DNA was quantified using 0.8% agarose gel electrophoresis and Qubit Fluorometer (Invitrogen, US). DNA concentrations were normalized to 100 ng/µl for subsequent library construction.

### Assessment of the genome assembly and annotation

**Fig. 4:**
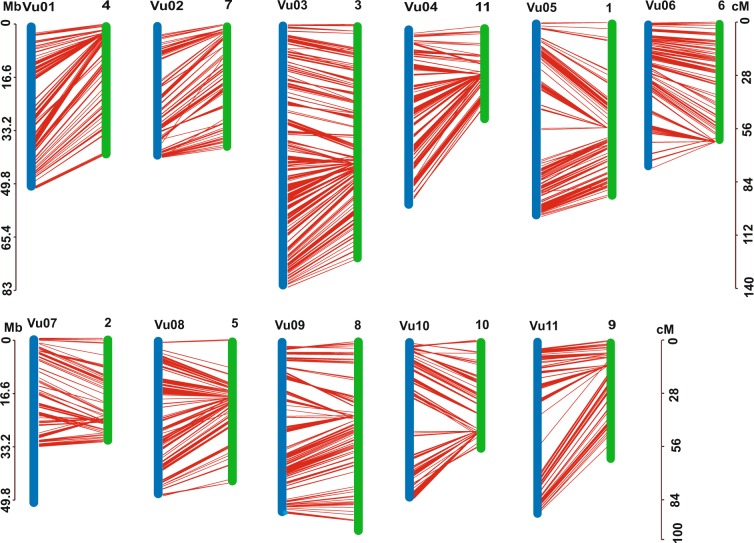
Synteny between asparagus bean pseudo-chromosomes and “ZZ v.2” linkage map. Each linkage on the right corresponds to one chromosome on the left with lines.

**Fig. 5:**
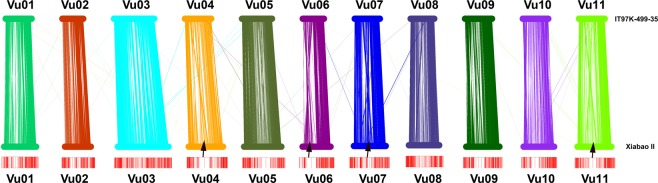
Comparative genome analysis between Xiabao II and IT97K-499-35^a^. Black arrows indicated the inconsistent areas between these two genomes.

Completeness of the genome assembly was assessed with default settings using the Benchmarking Universal Single-Copy Orthologs (BUSCO)^48^ approach with a total of 1440 orthologue groups from the Embryophyta Dataset. The results showed that 93.2% of the core orthologs could be found in the asparagus bean genome, indicating a high-integrity assembly superior to the other four legume genomes. We aligned the raw reads from short insert-size sequencing back to the assembly and showed that approximately 94.88% of short reads could be successfully mapped. Furthermore, a previously reported high-density linkage map (“ZZ” linkage map v.2)^5^ was used to assess the quality of anchored scaffolds. The sequences of 7,964 SNPs markers were aligned onto the 11 pseudo chromosomes using BLAT with parameters of “-fine”^49^. High accordance was shown between the assembled genome and the linkage map (Fig. 4). Whole genome comparative analysis was also conducted between this assembly and the cowpea genome available from Phytozome^11^ by the MUMmer with nucmer^50^, presenting high collinearities with four inconsistent areas, which are located in the low-recombination regions (Fig. 5).

### Comparison of asparagus bean genome with published common cowpea genomes

A comparison was performed (Table 11) between the asparagus bean genome and previously published common cowpea assemblies^8,10,11^. The asparagus bean genome assembly (549.81 Mb, non-N) was significantly larger than the first published IT97K-499-35^b^ genome^8^. Its size was close to the other three common cowpea survey assemblies (IT97K-499-35^a11^,IT97K-499-35^c^ and IT86D-1010)^10^. The scaffold N50 size of our asparagus bean genome was 2.7 Mb, longer than the other three genomes assembled by the next-generation sequencing technology. Moreover, the asparagus bean assembly had about 94% of the scaffolds anchored into 11 pseudo-chromosomes according to the high-density genetic map. In addition, a set of 42,287 common cowpea coding sequences (CDS) derived from the single molecule real-time technology^11^ could be blasted back to our asparagus bean genome with 90% similarity. All these results showed that the asparagus bean genome was of high quality.

**Table 11:** Comparisons of other four published cowpea assemblies.

	Xiabao II	IT97K-499-35^a^	IT97K-499-35^b^	IT97K-499-35^c^	IT86D-1010
Assembled Non-N Size (Mb)	549.81	518.8	323.3	568.1	609.5
GC content (%)	28.78	32.99	35.96	33.6	33.59
Repeat elements (%)	46.47	49.5	NA	NA	NA
Scaffold N50 size (kb)	2730.26	16,417.66	6.33	17.92	36.69
Total scaffolds	21,836	722	644,126	57,590	39,123
Number of Anchored into chromosomes	1,556	47	NA	NA	NA
Annotated protein-coding genes	42,609	29,773	NA	NA	NA
Numbers of CDS^d^	41,457	42,287	14,994	40,055	40,198

^a^IT97K-499-35 assembled by Lonardi *et al*. 2019.

^b^IT97K-499-35 assembled by Munoz-Amatriain *et al*. 2017.

^c^IT97K-499-35 assembled by Spriggs *et al*. 2018.

^d^A total of 4,2287 cds sequences from Vigna unguiculata v1.0, NSF, UCR, USAID, DOE-JGI, http://phytozome.jgi.doe.gov/.

## ISA-Tab metadata file

Download metadata file

## Data Availability

All tools used in this study were properly cited in the sections above. Settings and parameters were also clearly described.
